# Supplementary material for: Mechanisms governing the pioneering and redistribution capabilities of the non-classical pioneer PU.1
Source: Nat Commun. 2020 Jan 21;11:402. doi: 10.1038/s41467-019-13960-2 (PMC6972792; doi:10.1038/s41467-019-13960-2)
Supplement: Supplementary file 7 — Source data [file 41467_2019_13960_MOESM7_ESM.zip › Source_Data/FigureS2/FigureS2B_MotifScanOutput/KmeansCluster2/homerResults/motif14.similar.html]

motif14

## Information for motif14

T
C
A
G
A
T
G
C
T
C
G
A
C
G
T
A
C
T
A
G
C
T
A
G
A
C
T
G
C
T
A
G
A
T
C
G
C
G
T
A
G
T
C
A
  
Reverse Opposite:  

C
A
G
T
G
C
A
T
T
A
G
C
G
A
T
C
G
T
A
C
G
A
T
C
G
A
T
C
G
C
A
T
A
G
C
T
T
A
C
G
A
G
T
C
  

|  |  |
| --- | --- |
| p-value: | 1e-69 |
| log p-value: | -1.600e+02 |
| Information Content per bp: | 1.687 |
| Number of Target Sequences with motif | 362.0 |
| Percentage of Target Sequences with motif | 7.21% |
| Number of Background Sequences with motif | 886.2 |
| Percentage of Background Sequences with motif | 2.18% |
| Average Position of motif in Targets | 100.7 +/- 54.2bp |
| Average Position of motif in Background | 94.7 +/- 80.4bp |
| Strand Bias (log2 ratio + to - strand density) | 0.4 |
| Multiplicity (# of sites on avg that occur together) | 1.06 |
| Motif File: | file (matrix) reverse opposite |

### Similar de novo motifs found

|  |  |  |  |  |  |  |  |
| --- | --- | --- | --- | --- | --- | --- | --- |
| Rank | Match Score | Redundant Motif | P-value | log P-value | % of Targets | % of Background | Motif file |
| 1 | 0.905 | T C A G G A C T C A G T C G A T G A T C A G T C G A T C A G T C G A T C C G A T A C G T A C T G G T A C | 1e-58 | -134.686421 | 3.60% | 0.65% | motif file (matrix) |
| 2 | 0.901 | C T G A C T A G A T G C C T G A C T A G C A T G C T A G C T A G C T A G C A T G G C T A T C G A | 1e-44 | -103.190423 | 5.89% | 2.11% | motif file (matrix) |
| 3 | 0.850 | A C T G A T G C T C G A T C A G C T A G C T A G A C T G C T A G C T A G C G T A | 1e-34 | -80.515053 | 4.16% | 1.39% | motif file (matrix) |
| 4 | 0.773 | G A T C A G C T A G C T A T G C G T A C G T A C A G T C G A C T A G T C C G A T C A T G A T G C G A T C G C A T | 1e-34 | -78.986943 | 2.87% | 0.72% | motif file (matrix) |
